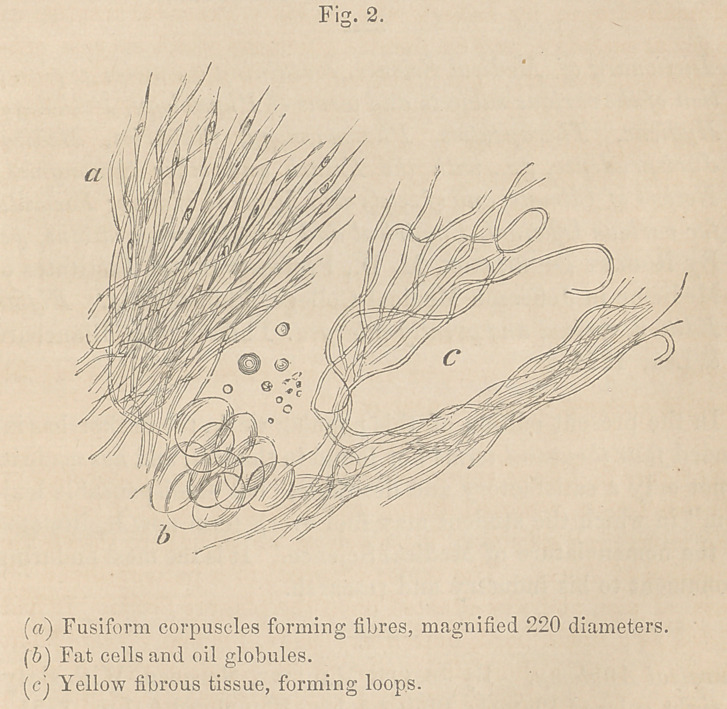# Microscopical Observations of Tumors

**Published:** 1851-11

**Authors:** John H. Brinton

**Affiliations:** Philadelphia


*Microscopical Observations of Tumors.* By John H. Brinton, of Philadelphia.

It is only since a comparatively recent period, that the microscope has been made applicable to the study of morbid growths, and although many observations have been made, and a number of excellent works published on this subject upon the continent of Europe, still it cannot yet be said, that the morphological arrangements of tumors, more especially of those which are classed under the general term malignant, have been sufficiently elucidated. The diagnosis of such growths has ever been an object of much study and interest to the Surgeon, and any means which can assist his diagnosis, or verify it subsequent to operation, deserve his most careful consideration. Is not the microscope subservient to this most important end? Inasmuch as this subject is beginning now to occupy the minds of the profession generally throughout this country, I shall make no apology for adding the records of the comparatively few cases I have had the opportunity of examining, to the general stock of information, in the certain expectation that at no distant day, the careful and continued investigation of this subject will be attended with results highly important, not only to pathologists generally, but more especially to the practical surgeon.

G. B., æt. 60 years, presented himself at the Surgical Clinic of Jefferson Medical College, complaining of hæmorrhoids. He stated that he had lost large quantities of blood, and was desirous of having the tumor removed. This was accordingly done by Prof. Pancoast, in the latter part of September, before the class. Ligatures were passed through the pedicle of the tumor, which was strangulated, and then removed.

The tumor was about the size of a small orange, soft to the touch, crushing down under the fingers, and very vascular. It had occupied only four weeks in attaining to its present size. The mucous membrane of the rectum, at the point at which the pedicle of the tumor was attached, felt hard to the touch, and scirrhous in its character.

Fig. 1.

(*a*) Cancer cells of various shapes and sizes, magnified 220 diameters showing single and double nuclei, and also granular matter.

(*b*) Arrangement of cells in layers.

(*c*) Action of acetic acid on cells.

Judging from external appearances, the tumor was apparently malignant, and its probable return was stated to the class. Upon squeezing out a little of the fluid of the tumor, and submitting it to the microscope, I found it to contain many cancer cells of different forms, oval, heart-shaped, elongated and round, the oval cells being most numerous, and the round least so. (Fig. 1, *a.*) The cells varied in size from the 1-1000th to the 1-2200th part of an inch in diameter, presenting a clear distinct outline; all were possessed of nuclei and nucleoli, and, in many cells, I found two nuclei. These nuclei were about the 1-4000th part of an inch in diameter. The interspace between the nucleus and the cell wall was filled with granular matter. Many molecules and granules were to be seen floating among the cells, but I did not observe the compound granular cell described by Bennett. Upon placing a portion of the more solid part of the tumor under the field of the microscope, the same appearance of the cells and of the granular matter was observed. The arrangement, however, of the cells was very peculiar and beautiful. They were enclosed in a net work of fibrous tissue, of extreme delicacy. (Fig. 1, *b.*) This tissue did not, as we often find it, enclose the cells in cysts, but in layers, one upon another. The addition of acetic acid rendered the cell wall transparent, but did not dissolve it, this is proved by the fact that if we drop iodine on the specimen, the cell is instantly colored, and the cell wall brought out definitely. The microscopical character of this tumor was the same as that usually presented by encephaloid.

I have used the term cancer cell, not meaning, however, to assert that we must consider any one form of cell as certainly and alone characteristic; indeed, in this very case we have seen several different forms of the cancer cell. That these different forms are, however, only modifications, is admitted by Bennett and insisted upon by Lebert, who, in his "Practical treatise on cancerous maladies, and on curable affections which may be confounded with cancer," published but two or three months since, says: "The type of the cancer cell is a small regular sphere with an elliptical nucleus, occupying about half of the interior of the cell, and containing one or more nucleoli, but that type is not often pure. The cellular envelope takes the ovoid, triangular heart and caudate shape. . . . . . . It would be useless to recount here all the shapes assumed by the cancer cell, it is sufficient to remark that in no other cell do we observe this *multiformity* of the cell wall to the same degree. . . . . The nucleus is, as we have already seen, the constant element of the cancer cell."

### Case II.

At figure 2, I have represented the microscopic appearance of a fibrous tumor, removed during the month of August last. It is an interesting specimen, inasmuch as we have here shown the formation of fibres. The tumor occurred in a patient about 60 years of age, and was situated upon the plantar fascia. It had existed as a small, hard, button-like body, for many years; but it was not until a short time prior to its removal, that it occasioned any inconvenience; it then began to enlarge rapidly, and attained nearly the size of a walnut. The tumor was closely adherent to the skin, which was with difficulty dissected off, and appeared to be of unusual thickness. Upon making a thin section of the tumor, which is best done by the use of Valentin's double-bladed knife, and placing it under the field of the microscope, the true character of the growth became apparent. It was observed to consist of a fibrous tissue, or rather of bands of tissue; the bands, in some instances running side by side, in a wavy manner; and in others interlacing one with another. The fibres, where separated by the use of the needle, appeared to partake of the character of yellow fibrous, or elastic tissue, forming loops. (Fig. 2, *c.*) Here and there might be seen the fusiform corpuscles described by Bennett and Lebert, which were composed of a simple nucleus, with two caudated extremities, the nucleus being about the 1-3000th part of an inch in diameter. These fusiform corpuscles, by their juxtaposition, evidently went to the formation of fibres.

Fig. 2.

(*a*) Fusiform corpuscles forming fibres, magnified 220 diameters.

(*b*) Fat cells and oil globules.

(*c*) Yellow fibrous tissue, forming loops.

Fat vesicles, and free fat globules were to be found in abundance throughout the whole tumor. (*b.*)

The fusiform corpuscle, the caudate cancer cell of the older microscopists, has given rise at different times to much dispute; although at one period considered to be characteristic of cancer.

It has since been shown that we must expect to find it in all growths where fibrous tissue is at all involved. It is owing to this circumstance that in scirrhus we almost always have it, and we often detect it going to the formation of the reticulum of encephaloid, but no dependence can be placed on its presence as a mark of malignant growth.

## Figures and Tables

**Fig. 1. f1:**
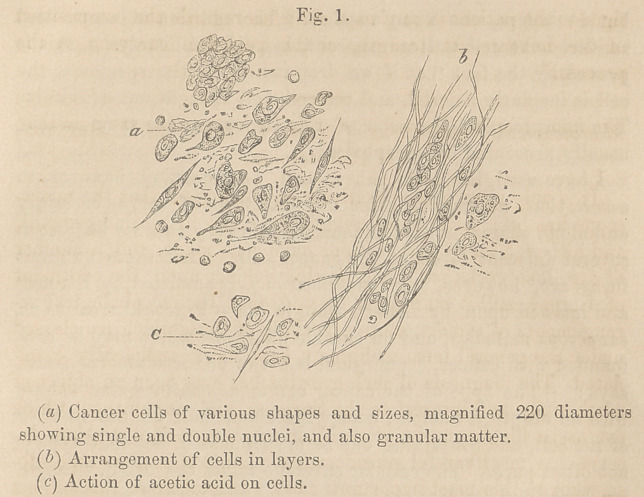


**Fig. 2. f2:**